# Molecular Profiling and Treatment Outcomes in Uterine Serous Carcinoma: Prognostic Role of Estrogen Receptor Expression

**DOI:** 10.3390/curroncol33030132

**Published:** 2026-02-24

**Authors:** Anna Svarna, Michalis Liontos, Kallirroi Goula, Konstantina Pardali, Konstantinos Koutsoumpogeras, Katerina Aravantinou, Konstantina Christina Perdikari, Ioanna Kollarou, Maria Kaparelou, Dimitrios Haidopoulos, Constantine Dimitrakakis, Meletios Athanasios Dimopoulos, Flora Zagouri

**Affiliations:** 1 Department of Clinical Therapeutics, National and Kapodistrian University of Athens, Alexandra Hospital, 11528 Athens, Greece; mlionto@med.uoa.gr (M.L.); fzagouri@med.uoa.gr (F.Z.); 2 Department of Pathology, Alexandra Hospital, 11528 Athens, Greece; 3 Department of Medical Oncology, Areteio Hospital, 11528 Athens, Greece; 4 Obstetrics and Gynecology, National and Kapodistrian University of Athens, 1st Obstetrics and Gynecology Clinic, 11526 Athens, Greece; 5 Department of Medicine, Korea University, Seoul 02841, Republic of Korea

**Keywords:** uterine serous carcinoma, endometrial cancer, molecular classification, estrogen receptor, TP53 mutation, HER2 amplification, prognostic biomarkers

## Abstract

Background: Uterine serous carcinoma (USC) represents a rare but aggressive subtype of endometrial cancer, accounting for a disproportionate number of disease-related deaths. Although molecular classification has improved risk stratification, prognostic heterogeneity highlights the need for new prognostic markers. Methods: We retrospectively analyzed 83 patients with USC treated at our institution between 1 January 2015 and 31 December 2023. Clinicopathological characteristics, treatment strategies, molecular biomarkers accessed by immunohistology (TP53, ER, PR, HER2, and MMR status), and survival outcomes were collected. Patients were first staged by FIGO 2009 and retrospectively reclassified by FIGO 2023. Disease-free survival (DFS), progression-free survival (PFS), and overall survival (OS) were assessed using Kaplan–Meier and Cox regression analyses. Results: The majority of patients were presented with advanced disease (FIGO stage IIIC-IV). TP53 mutations were found in 88% of cases, HER2 amplification in 18%, and ER expression in 57.8%. ER-positive patients showed significantly improved DFS in the adjuvant setting compared with ER-negative patients, whereas no significant associations were observed for first-line PFS or OS in multivariable analyses. HER2 amplification was not associated with inferior survival in our cohort. The advanced stage remained an independent predictor of worse OS. Conclusions: USC is a biologically heterogeneous disease, and its treatment should be guided by its molecular profile. ER expression identifies a subset of patients with improved DFS, suggesting potential prognostic relevance in this high-risk histology.

## Simple Summary

Uterine serous carcinoma is a rare and aggressive form of endometrial cancer often diagnosed at an advanced stage. In this retrospective single-institution study, we analyzed the clinical characteristics, treatments, molecular markers, and outcomes of 83 patients. Most tumors showed TP53 mutations, while estrogen receptor (ER) expression and HER2 amplification were also observed. ER-positive tumors were associated with improved disease-free survival after initial treatment, although no association was observed with progression-free or overall survival. These results highlight the biological heterogeneity of uterine serous carcinoma and the potential prognostic role of ER expression in this population.

## 1. Introduction

Endometrial cancer is the most common gynecological malignancy in developed countries, with more than 69,000 women expected to be diagnosed in 2025 in the United States alone. Endometrioid carcinomas make up the majority of endometrial cancers; however, in about 10–20% of cases, the histology reveals a uterine serous carcinoma (USC). Although rare, USCs account for 40% of endometrial cancer deaths, holding a worse prognosis status, being diagnosed at a more advanced stage, and often recurring outside the pelvis [1,2,3].

The comprehensive molecular analysis performed by TCGA has fundamentally changed our understanding of the neoplasm and its classification. Historically, USCs were categorized as type 2 endometrial cancers, being non-endometrioid and high-grade according to their histology. Now, we recognize four subtypes with specific molecular characteristics: POLE ultra-mutated, microsatellite instability hyper-mutated (dMMR), copy-number low, and copy-number high [4]. However, in daily practice, it is more common and accessible to test for the surrogate markers of these subgroups (e.g., p53 immunohistochemistry, microsatellite instability, and POLE proofreading mutation), which is a practical approach to apply molecular classification. Hence, molecular classification plays a vital role in the latest FIGO 2023 staging [5], where patients are stratified into risk groups that inform their need for therapy in early stages.

In line with the molecular classification and the new FIGO 2023 staging system, new guidelines were introduced in 2025. In these, the copy-number low or no specific molecular profile (NSMP) patients were reclassified according to estrogen receptor expression and grade into NSMP low-grade and estrogen receptor-positive and NSMP high-grade or estrogen receptor-negative (or both) [6]. The change was made due to the different biological behavior of the two subgroups that can inform clinical practice. In the new 2025 system, adjuvant treatment is guided by both tumor stage and the four molecular subgroups (POLEmut, dMMR, NSMP low-grade and estrogen receptor-positive, NSMP high-grade or estrogen receptor-negative (or both), TP53 mutant (p53mut)) [6].

The use of the molecular classification also produced informative results in the recent phase III trials, adding immunotherapy to first-line chemotherapy. As anticipated, dMMR/MSI-high patients showed a clear and substantial benefit from the addition of immunotherapy across all trials. Focusing on the p53mut subgroup, however, and knowing that it is mostly comprised of a serous histology, the results were mixed [7,8,9]. That discordance of histological and molecular classification indicates that further investigation is essential to clarify the complexities associated with this neoplasm.

Serous endometrial carcinomas, though mostly p53mut, are a heterogeneous subgroup. Human epidermal growth factor receptor 2 (HER2) overexpression/ERBB2 amplification has also been associated with USC in about 20%, depending on the method used and the patient series [10]. Moreover, a minority harbors a targetable genetic mutation like BRCA1/2 or PIK3CA [11]. A more in-depth look into the molecular peculiarities of USC is thus needed to further identify new prognostic factors and predictive factors that will improve patient management and therapeutic outcomes.

The goal of this study is to showcase serous endometrial cancer patients’ characteristics, to follow patients’ treatment journey, and, through the implementation of the molecular classification, to explore the emergence of new prognostic factors.

## 2. Methods

### 2.1. Patients

**Figure 1:**
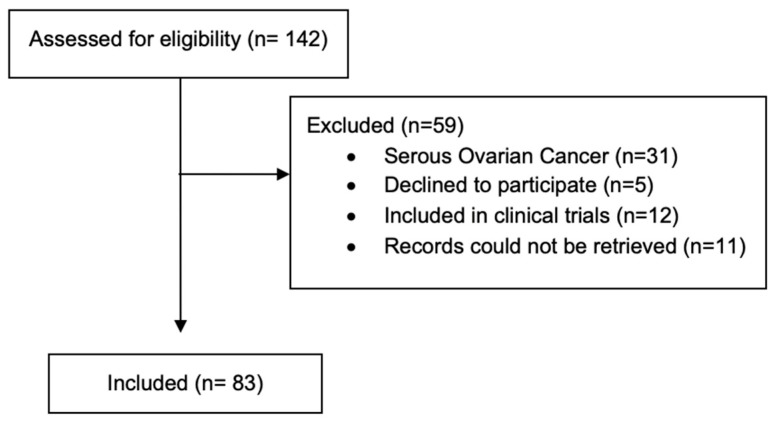
Patient consort diagram.

We included patients aged 18 years or older with a confirmed diagnosis of USC who were diagnosed after 1 January 2015 and treated in our Oncology unit. All patients provided written informed consent for the use of their medical records for research purposes. Our Institutional Review Board approved the study and it was conducted according to the Declaration of Helsinki. Patients enrolled in clinical trials were not included, nor were patients whose data could not be retrieved from our records. The consort diagram showcases the inclusion of the patients prosses (Figure 1).

Clinicopathological, treatment-related, and survival data were collected through a single-institution database. The data collected included patient demographics (age and ECOG performance status), clinical characteristics (histological subtype, tumor stage at diagnosis, and the presence of metastases), treatment regimen details (surgery, adjuvant treatment, and the treatment of metastatic/recurring disease), and survival data. Tumor staging was performed following the FIGO 2009 classification system for the data to be comparable throughout the years. Disease progression was defined, according to the Response Evaluation Criteria in Solid Tumors (RECIST), as a new metastatic lesion and/or an ≥20% increase in the sum of diameters of target lesions and/or clinical deterioration. Death was not assessed as being cancer-related or non-cancer-related.

### 2.2. Molecular Characterization

An immunohistochemical study was conducted to analyze ER, PR, HER2, and TP53 expression. Specifically, monoclonal antibodies Rabbit estrogen-clone EP1 were used to determine ER expression and a 10% cut-off was used for ER positivity, monoclonal mouse progesterone-clone PgR 636 for PR expression, and polyclonal rabbit cerbB2-oncoprotein for HER2-cerbB2 expression.

To determine the MMR status, a subsequent immunohistochemical expression study of the proteins associated with microsatellite instability was performed. Monoclonal mouse antibodies Homolog1 clone EP45 were used for MLH1 expression, monoclonal mouse Homolog2 clone FE 11 for MSH2 expression, monoclonal rabbit Homolog6 clone EP45 for MSH6 expression, and monoclonal rabbit increased 2 clone EP51 for PMS2 expression. Patients with complete expression of the above proteins were classified as MMR proficient or MS-stable, while those lacking expression were classified as MMR deficient or MSI-high.

### 2.3. Statistical Analysis

Descriptive statistics were used to summarize patient characteristics. Continuous variables were reported using the median and interquartile range (25th–75th percentiles), whereas categorical variables were presented as absolute frequencies and percentages. Survival outcomes, including overall survival (OS), disease-free survival (DFS), and progression-free survival (PFS), were analyzed. OS was defined as the time from initial diagnosis to death from any cause or last follow-up. PFS was calculated from the time of diagnosis of advanced or recurrent disease to disease progression or death from any cause.

Survival probabilities were estimated using the Kaplan–Meier method, and differences between groups were assessed with the log-rank test. Univariable and multivariable Cox proportional hazards regression models were applied to estimate hazard ratios (HRs) with corresponding 95% confidence intervals (CIs). All statistical tests were two-sided, and a *p*-value < 0.005 was considered statistically significant. Statistical analyses were performed using SPSS software v31.0.0.

## 3. Results

### 3.1. Patients’ Characteristics

Between 1 January 2015 and 31 December 2023, 83 patients with serous endometrial cancer were identified and their clinical data analyzed. The last patient follow-up was conducted on 8 December 2025 to allow for a median follow-up time of 57.0 months. The median age at diagnosis was 66.1 years (25th–75th percentile: 62.0–71.9 years). Their clinicopathological characteristics and demographics are presented in Table 1. In this series of 83 patients, almost two-thirds presented with advanced FIGO stages (IIIC–IVB), revealing the aggressive nature of the disease. Mixed histology, mainly with endometrial carcinoma, accounted for 15.5% of the patients. Seventy-four patients underwent surgery, with the majority of them having lymphadenectomy (49/74) and omentectomy (61/74).

All patients in our cohort were staged using FIGO 2009, as it was the available staging system at the start of our data collection. However, we retrospectively restaged patients using the FIGO 2023 staging using both the clinicopathological and the molecular data collected. The change in our patients’ staging is shown in Supplementary Figure S1.

### 3.2. Immunohistochemical Analysis

**Figure 2:**
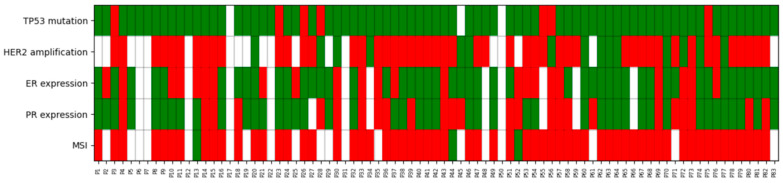
Molecular biomarker heatmap. Heatmap of TP53, HER2, ER, PR, and MMR status across the patient cohort. Green indicates: TP53-mut, HER2 amplification, ER or PR expression, and dMMR status. Red indicates: TP53-wt, no HER2 amplification, no ER or PR expression, and pMMR status, and white indicates missing results. Rows represent molecular markers and columns represent individual patients.

We performed a biomarker analysis retrospectively in order to further classify uterine serous carcinomas according to the most recent molecular classification. POLE mutation analysis was not performed since only endometrioid neoplasms have been shown to bear such mutations. The data on the analysis of MMR proteins, Hormone Receptors (ER and PR), HER2 expression, and TP53 mutation assessed by Immunohistochemistry, are displayed in Table 2, and a visual heatmap of the molecular profile of each patient is shown in Figure 2. Moreover, we performed a correlation analysis (Chi-square) between ER status and FIGO Stage, and no significant association was observed between estrogen receptor status and FIGO stage (χ^2^ test, *p* = 0.239). A more comprehensive comparison between ER-positive and ER-negative patient characteristics can be found in the Supplementary Materials. Due to high missing rates in the MMR and HER2 status, a comparison of the patients can be found in the Supplementary Materials showing the selection bias.

### 3.3. Adjuvant Treatment

In total, 47 patients underwent optimal surgery and were eligible for adjuvant treatment; among them, 44 received treatment, as one had prior neoadjuvant treatment and 2 rapidly progressed. The exact adjuvant regimen that they received is shown in Table 3. Most patients (42/44) received chemotherapy; 36/44 received chemotherapy in combination with a radiotherapy modality; and 25 received a combination of EBRT and brachytherapy.

Of the patients with no evidence of disease after surgery, 30/47 progressed; 18/30 (60%) with new metastatic disease, 7/30 (23.3%) experienced a clinical progression (defined as a clinical deterioration related to disease before radiographic progression and death), and 5/30 (16.7%) had both a clinical deterioration and documented new metastasis.

### 3.4. Treatment for Metastatic Disease

A total of 59 patients received first-line treatment in our cohort. Data on the site of disease recurrence were available for 57 of them. Recurrence within the pelvis was the most prominent pattern among patients analyzed (24 patients, 42.1%), followed by those who had multiple metastatic sites—within and outside of the pelvis (22 patients, 38.6%). In 11 patients (19.3%), disease recurrence occurred only at sites outside the pelvis. The exact regimen received in first-line treatment is shown in Table 4.

Thirty-three patients received the combination of Carboplatin and Paclitaxel, six in combination with immunotherapy, and eight in combination with trastuzumab following HER2 positivity results. Patients who received different platinum doublets and the combination of pembrolizumab plus lenvatinib were those who progressed after adjuvant treatment. Notably, no patient received hormonal therapy in first-line treatment.

**Figure 3:**
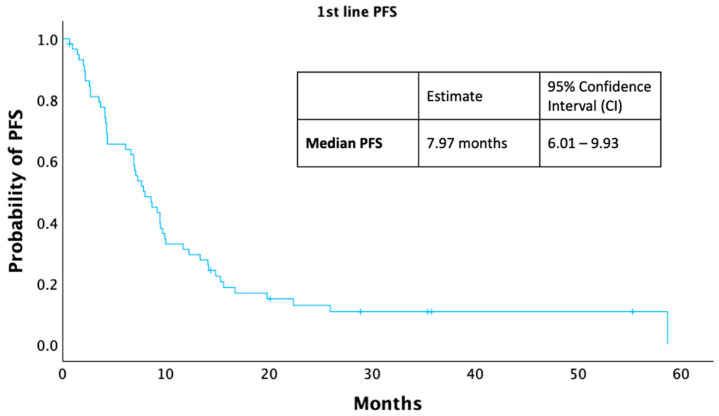
Kaplan–Meier curve depicting first-line PFS.

A total of 52 (88.1%) of the 59 patients who received first-line treatment progressed. Among these, the most common pattern of disease progression was the appearance of new metastatic sites (16/52). Progression, defined as an increase in lesion size, occurred in 12/52 patients, while clinical progression (based on symptoms, performance decline, or death) occurred in another 12/52. Finally, an additional 12/52 patients exhibited more than one type of progression (e.g., both radiologic and clinical). In Figure 3, the patients’ median progression-free survival (mPFS) in this setting was 7.9 months (95% Cl: 6.01–9.93).

### 3.5. Subsequent Therapies

Treatment data were available for 38 patients in the second-line setting and 22 patients in the third-line setting, and are displayed in Supplementary Table S1.

The most commonly used regimen in the second-line landscape was Pembrolizumab and Lenvatinib (15 patients, 39.5%), followed by platinum-based doublets (10 patients, 26.3%) and Doxorubicin (4 patients, 10.5%).

A total of 35 patients progressed on second-line treatment, but only 22 received a subsequent therapy. Third-line treatment regimens displayed greater heterogeneity, including Paclitaxel monotherapy (eight patients, 36.4%), Trastuzumab Deruxtecan (four patients, 18.2%) for HER-2 positive cases, and platinum doublets (four patients, 18.2%). Fifteen of the 20 patients who progressed in third-line treatment also received a fourth line of therapy. A small proportion were enrolled in a clinical trial in that setting (2 cases, 9.1%). The treatment lines received by our patients are shown in Supplementary Figure S2.

### 3.6. Multivariate Analysis

A multivariate analysis was conducted to further delineate independent predictors of unfavorable survival outcomes. Patients’ characteristics, including age at diagnosis, stage at diagnosis, HER-2 expression, MMR status, and ER status, were included. The analysis was conducted regarding DFS in patients receiving adjuvant therapy, PFS among those receiving first-line treatment, and OS in the entire population (Table 5).

## 4. Discussion

Endometrial cancer represents a biologically heterogeneous disease, encompassing tumors with distinct molecular profiles, clinical behavior, and therapeutic vulnerabilities. Although molecular classification has substantially improved prognostic stratification and treatment individualization, considerable outcome variability persists even within defined molecular subgroups. This variability highlights the ongoing need for additional biomarkers capable of refining prognostic subgroups and identifying novel therapeutic targets, particularly in histologic subtypes traditionally associated with aggressive behavior and limited treatment options.

In this context, emerging evidence suggests that ER expression may show prognostic relevance even in high-risk histologic subtypes, including serous endometrial carcinomas. While serous tumors are classically considered hormone receptor–negative and biologically aggressive, a subset demonstrates ER expression, raising the possibility that hormonal differentiation may identify a group with distinct clinical behavior. These findings support the hypothesis that ER expression could contribute to prognostic stratification beyond conventional histologic and molecular features, warranting further investigation of its clinical significance in serous endometrial cancers.

ER positivity in our patients was high at 57.8% (48/83). Despite this being considered a pathological characteristic of endometrioid endometrial cancers, the expression of hormone receptors is indeed quite frequent in USC and even reported as high as 65% [12]. Most patients, however, did not have that result during diagnosis, and none of them received hormonal therapy due to the aggressiveness of the serous histology.

ER positivity has changed the treatment landscape for low-grade NSMP patients, identifying a subgroup of good-prognosis patients who can de-escalate adjuvant treatment according to the new 2025 guidelines [6]. Even in the dMMR population, there is a sign that ER positivity leads to better outcomes. In our cohort, we explored the prognostic value of ER positivity in this inherently high-risk histological subgroup. We found that ER-positive patients had better DFS, but that advantage was lost in the first-line PFS and the overall survival multivariate analysis. This discrepancy may be attributed to the small number of patients in our cohort, but it definitely deserves further investigation in order to understand the interplay between different molecular pathways in patients with USC.

The role of hormone receptors in the prognosis of endometrial cancer has been thoroughly studied; however, their role in serous carcinomas in light of the new molecular classification is inadequately reported. Although not unanimously, the literature has shown that hormone receptor-positive endometrial neoplasms are associated with better survival [13,14,15,16]. We believe our results bridge the gap between literature based on histological subtypes and the new molecular classification.

The molecular classification in our cohort did not include POLE NGS testing, as POLE mutations are extremely rare in serous histology and, consequently, POLE sequencing is not routinely done in daily clinical practice [17]. As expected, the majority of patients exhibited a p53mut profile, consistent with previously published data [18]. It should be noted that TP53 was evaluated by immunohistochemistry (IHC) as recommended by the guidelines [6]; however, none of the results were cross-validated using gene sequencing.

Only 8.4% of our patients demonstrated a TP53 wildtype (p53wt) pattern. The small sample size that was determined as p53wt did not allow us to properly explore the role of TP53 in this subgroup. The clinical behavior of this subpopulation has been shown to be more indolent than that of p53mut patients, suggesting a need for further research to clarify the prognostic role of TP53 in this histology subgroup [19].

Moreover, only three patients were retrospectively found to be dMMR; all three were p53mut, as per our heatmap, and were therefore labeled as double classifiers. All were diagnosed at stage IIIc and received adjuvant treatment with chemotherapy (2/3) and a combination of chemotherapy and radiation (1/3). Only one progressed, and none received immunotherapy. Bibliography on dMMR p53mut USC is scarce, and according to the 2025 guidelines, it is to be classified as dMMR, as it is believed to be its genomic driver and correlates better with the clinical course of the disease [6].

Regarding HER2-positive patients, as trials targeting HER2-positive disease were available at the time, most were tested concurrently with their diagnoses. According to the literature on USC patients, HER2 gene amplification is associated with worse disease clinicopathological characteristics, such as higher stage [20,21,22] and higher relapse rates in localized disease [23]. In our cohort, HER2 positivity was not a prognostic factor for worse DFS. We believe this can be partly explained by the higher stage of diagnosis of these patients, but warrants further investigation.

However, this dismal prognosis can be changed by HER-2 targeting therapies. A total of 10 out of the 15 HER-2 amplified patients in our cohort received targeted therapy, either trastuzumab combined with chemotherapy in the frontline setting [24], or trastuzumab deruxtecan in subsequent lines based on the results of the DESTINY-PanTumor 02 study [25]. Perhaps these targeted therapies altered the disease course and obscured the worst prognosis these patients have in other studies, explaining the lack of prognostic value of HER2 in the multivariate analysis of first-line and OS.

Furthermore, we analytically showcased the treatments our patients received. The treatment algorithm consisted of the combination of paclitaxel/carboplatin with or without targeted therapy or immunotherapy, followed by the doublet of immunotherapy and lenvatinib if immunotherapy had not already been given. It aligns with the guidelines at the time; however, the high percentage of other therapies (monotherapy and other platinum doublets) underscores the treatment uncertainty for these high-risk patients and the urgent need for personalized care. Lastly, the addition of targeted HER2 therapies in the HER2-positive population, we believe, changed our patients’ clinical course.

The characteristics of the patients in our cohort are consistent with previously published data, particularly regarding the tendency to diagnose at more advanced stages of disease [26]. This alignment with the existing literature reinforces the representativeness of our sample. Moreover, the type of surgery performed reflects contemporary clinical practice, which has evolved to include lymphadenectomy or sentinel lymph node excision. Finally, the transition from the 2009 to the 2023 staging system provided a more refined stratification of disease, resulting in a staging framework that more accurately correlated high-risk patients with more advanced pathological stages.

This study has several limitations that should be acknowledged. First, POLE sequencing was not performed, which may have led to incomplete molecular characterization, particularly given the established prognostic significance of POLE-ultramutated tumors. Furthermore, not all patients underwent full molecular classification, potentially limiting the validity of our subgroup analyses. This is also the case for the wide confidence intervals noted in our multivariate analysis, but that was not related to the statistically significant findings, including the prognostic significance of ER expression in patients undergoing adjuvant treatment.

Despite these constraints, the study also presents important strengths. It is based on single-institution data, ensuring consistency in diagnostic and treatment pathways. The number of included patients is comparable to other published cohorts focusing on serous endometrial carcinoma, a relatively uncommon histologic subtype. Importantly, to the best of our knowledge, this is the first study to investigate the prognostic relevance of ER positivity within a current serous endometrial cancer population that has undergone molecular classification and has been staged according to the most recent staging system, thereby providing novel insights into an area with limited existing data.

## 5. Conclusions

In this single-institution cohort, molecular profiling confirmed the predominance of p53-mutant disease among USC while also identifying an ER-positive subpopulation associated with improved DFS in the adjuvant setting. Although this benefit did not extend to PFS or OS, these findings highlight the importance of integrating molecular and biomarker data and support further prospective research on hormone receptor expression to refine prognostic stratification and guide personalized management of uterine serous carcinoma.

## Figures and Tables

**Table 1:** Clinicopathological patients’ characteristics.

	Category	Number (*N*)	Percent (%)
**Performance Status (ECOG-PS)**	0	57	68.7
	1	11	3.3
	2	6	7.2
	Missing	9	10.8
**Mixed Histology**	No	71	85.5
	Yes	12	14.5
**Surgery**	No	9	10.8
	Yes	74	89.2
**Lymphadenectomy**	No	25	30.1
	Yes	49	59.0
	Not applicable	9	10.8
**Omentectomy**	No	14	16.9
	Yes	60	72.3
	Not applicable	9	10.8
**Stage (FIGO 2009)**	IA	10	12.0
	IB	9	10.8
	II	3	3.6
	IIIA	4	4.8
	IIIB	1	1.2
	IIIC	20	24.1
	IVA	18	21.7
	IVB	18	21.7

**Table 2:** Molecular data.

Biomarker	Category	Number (*N*)	Valid Percent (%)
**MMR**	Proficient	61	73.5
	Deficient	3	3.6
	Missing	19	22.9
**HER2 amplification**	Negative	49	59.0
	Positive	15	18.0
	Missing	19	23.0
**TP53 status**	Wild-type	7	8.4
	Mutant	73	88.0
	Missing	3	3.6
**ER**	Negative	23	27.7
	Positive	48	57.8
	Missing	12	14.5
**PR**	Negative	26	31.3
	Positive	45	54.2
	Missing	12	14.5

ER, estrogen receptor; PR, progesterone receptor.

**Table 3:** Adjuvant treatment received.

Treatment Type	Frequency (*N*)	Percent (%)
Chemotherapy Only	6	13.6
Chemotherapy + EBRT	2	4.5
Chemotherapy + EBRT + Brachytherapy	25	56.8
Chemotherapy + Brachytherapy	9	20.5
EBRT Only	1	2.3
EBRT + Brachytherapy	1	2.3

EBRT: External beam radiotherapy.

**Table 4:** Regimens used as first-line treatment.

Regimen	Frequency (*N*)	Percent (%)
Carboplatin/Paclitaxel	29	49.2
Paclitaxel/Carboplatin and ICIs	6	10.2
Paclitaxel/Carboplatin and Trastuzumab	8	13.6
Platinum Doublet (Non-Paclitaxel)	2	3.4
Carboplatin Monotherapy	3	5.1
Radiation Therapy	4	6.8
Other Regimens	4	6.8
Pembrolizumab and Lenvatinib	3	5.1

ICIs: Immune Checkpoint Inhibitors.

**Table 5:** Multivariant analysis for DFS, first-line PFS, and OS.

Variable	Adjuvant DFS			1st Line PFS			Overall Survival (OS)		
	*p*-Value	HR	95% CI	*p*-Value	HR	95% CI	*p*-Value	HR	95% Cl
**HER-2 EXPRESSION (0/1+/2+ CISH(-) VS. 3+/2+ CISH (+))**	0.107	2.986	0.791–11.276	0.453	0.741	0.339–1.662	0.288	0.582	0.215–1.579
**STAGE (IA-IIA, IIB-IIIC, IV)**	0.043	3.545	1.044–12.041	0.448	1.249	0.704–2.217	0.003	2.650	1.398–5.022
**ER EXPRESSION (ABSENT VS. PRESENT)**	0.046	0.339	0.115–0.979	0.303	1.526	0.683–3.407	0.723	0.872	0.409–1.858
**AGE**	0.012	1.105	1.022–1.194	0.463	0.977	0.919–1.039	0.839	1.398	0.955–1.059
**dMMR STATUS (PRESENT VS. ABSENT)**	0.934	1.092	0.136–8.760	0.230	3.6339	0.441–30.030	0.915	0.896	0.119–6.767

ER, estrogen receptor; dMMR, MisMatch Repair deficient.

## Data Availability

Data is unavailable due to privacy issues. The data presented in this study are available on request from the corresponding author.

## Supplementary Materials

The following supporting information can be downloaded at: https://www.mdpi.com/article/10.3390/curroncol33030132/s1, Figure S1: Stage migration FIGO 2009 to FIGO 2023; Table S1: Regimens used as 2nd and 3rd line treatment; Figure S2: Sankey diagram depicting patient transitions across successive lines of systemic therapy (L1–L4); Table S2: Clinicopathological characteristics according to HER2 and MMR data availability; Table S3: Clinicopathological characteristics according to ER status.
