# Supplementary material for: Molecular Profiling and Treatment Outcomes in Uterine Serous Carcinoma: Prognostic Role of Estrogen Receptor Expression
Source: Curr Oncol. 2026 Feb 24;33(3):132. doi: 10.3390/curroncol33030132 (PMC13025451; doi:10.3390/curroncol33030132)
Supplement: Supplementary file 1 [file curroncol-33-00132-s001.zip › curroncol-4127903-supplementary.pdf]

Supplemental material

Supplemental Figure S1 Stage migration FIGO 2009 to FIGO 2023

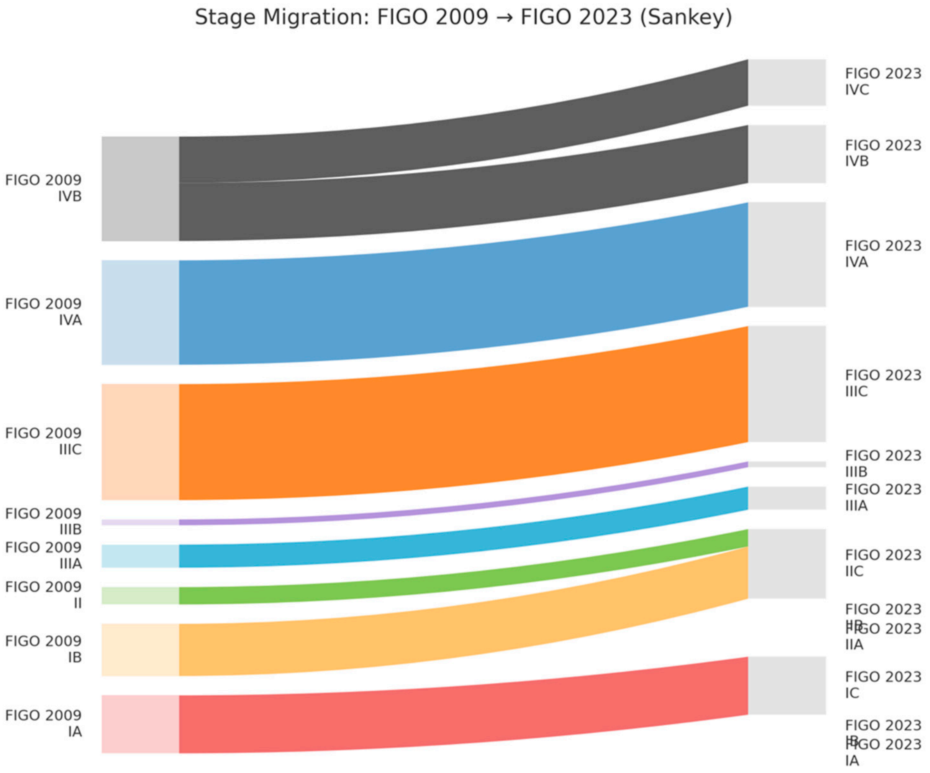

Supplemental Table S1. Regimens used as2nd and 3rd line treatment

Regimen	2nd Line	%	3rd Line	%
Carboplatin/ Paclitaxel	0	0.0	1	4.5
Pembrolizumab and Lenvatinib	15	39.5	0	0.0
Platinum Doublet (Non-Paclitaxel)	10	26.3	4	18.2
**PACLITAXEL /CARBOPLATIN** and ICI	1	2.6	0	0.0
Doxorubicin	4	10.5	1	4.5
Paclitaxel	2	5.3	8	36.4
Trastuzumab Deruxtecan	1	2.6	4	18.2
Radiation Therapy	1	2.6	0	0.0
Other Regimens	4	10.5	2	9.1
Tota	38	100	22	100

Supplemetal Figure S2 Sankey diagram depicting patient transitions across successive lines of systemic therapy (L1–L4)

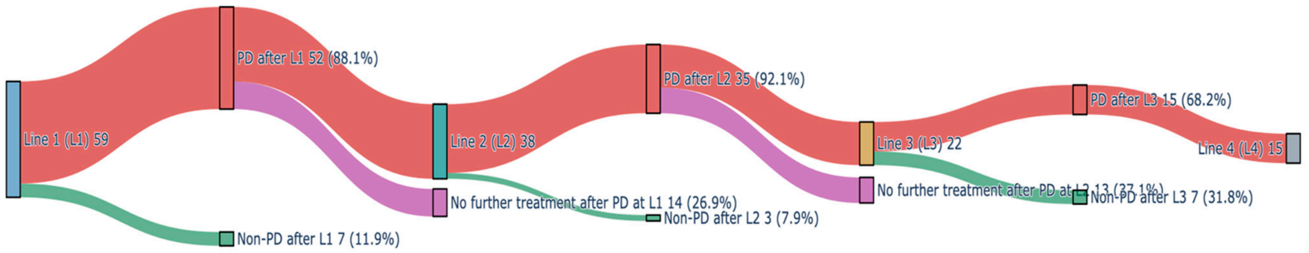

At each line, patients are categorized as having progressive disease (PD) or non–progressive disease (non-PD). Patients with PD may proceed to a subsequent line of therapy or discontinue treatment, whereas non-PD patients do not transition further. Flow widths are proportional to patient numbers, and percentages are calculated per line of therapy.

Supplemental Table S2 Clinicopathological characteristics according to HER2 and MMR data availability

		**HER 2**			**MMR**		
		Non-missing N=64	Missing N=19	p-value	Non-missing N=64	Missing N=19	p-value
	Category						
**Performace Status (ECOG-PS)**							
	0	46 (71.9%)	11 (57.9%)		44 (68.8%)	13 (68.4%)	
	1	9 (14.1%)	2 (10.5%)		9 (14.1%)	2 (10.5%)	
	2	4 (6.2%)	2 (10.5%)		4 (6.2%)	2 (10.5%)	
	Missing	5 (7.8%)	4 (21.1%)		7 (10.9%)	2 (10.5%)	
**Mixed Histology**				0.005			0.026
	No	59 (92.2%)	12 (63.2%)		58 (90.6%)	13 (68.4%)	
	Yes	5 (7.8%)	7 (36.8%)		6 (9.4%)	6 (31.6%)	
**Surgery**				0.264			0.264
	No	7 (10.9%)	4 (21.1%)		7 (10.9%)	4 (20.1%)	
	Yes	57 (89.1%)	15 (78.9%)		57 (89.1%)	15 (78.9%)	
**Lymphadenectomy**				0.072			0.408
	No	18 (28.1%)	7 (36.8%)		19 (29.7%)	5 (26.3%)	
	Yes	41 (64.1%)	8 (42.1%)		39 (60.9%)	10 (52.6%)	
	Not applicable	5 (7.8%)	4 (21.1%)		5 (7.8%)	4 (21.1%)	
**Omentectomy**				0.005			0.123
	No	7 (10.9%)	7 (36.8%)		8 (12.5%)	5 (26.3%)	
	Yes	52 (81.2%)	8 (42.1%)		50 (78.1%)	10 (52.6%)	
	Not applicable	5 (7.8%)	4 (21.1%)		5 (7.8%)	4 (6.3%)	
**Stage (FIGO 2009)**				0.094			0.206
	IA	8 (12.5%)	2 (10.5%)		8 (12.5%)	2 (10.5%)	
	IB	6 (9.4%)	3 (15.8%)		6 (9.4%)	3 (15.8%)	
	II	3 (4.7%)	0 (0%)		3 (4.7%)	0 (0%)	

	IIIA	1 (1.6%)	3 (15.8%)	1 (1.6%)	3 (15.8%)
	IIIB	1 (1.6%)	0 (0%)	1 (1.6%)	0 (0%)
	IIIC	16 (25.0%)	4 (21.1%)	16 (25.0%)	4 (21.1%)
	IVA	17 (26.6%)	1 (5.3%)	16 (25.0%)	2 (10.5%)
	IVB	12 (18.8%)	6 (31.6%)	13 (20.3%)	5 (26.3%)

Data are presented as n (% per column). HER2 and MMR non-missing indicate cases with available molecular testing results, whereas missing indicates unavailable data. ECOG-PS refers to baseline performance status. FIGO staging based on the 2009 classification.

Supplemental Table S3 Clinicopathological characteristics according to ER status

	ER-positive N (%)	ER-negative N (%)	P-value
**ECOG-PS (baseline)**			0.755
0	39 (66.1%)	20 (74.1%)	
>=1	12 (20.3%)	4 (14.8%)	
Missing	8 (13.6%)	3 (11.1%)	
**Mixed histology**			0.931
Yes	13 (22.0%)	5 (18.5%)	
No	46 (78.0%)	22 (81.5%)	
Missing	0 (0.0%)	0 (0.0%)	
**Surgery**			0.553
Yes	53 (89.8%)	26 (96.3%)	
No	6 (10.2%)	1 (3.7%)	
**Lymphadenectomy**			0.092
No	24 (40.7%)	6 (22.2%)	
Yes	29 (49.2%)	20 (74.1%)	
Not applicable	6 (10.2%)	1 (3.7%)	
**Omentectomy**			0.595
No	12 (20.3%)	6 (22.2%)	
Yes	41 (69.5%)	20 (74.1%)	
Not applicable	6 (10.2%)	1 (3.7%)	
**Stage (figo 2009)**			0.239
Advanced	42 (71.2%)	15 (55.6%)	
Early	17 (28.8%)	12 (44.4%)	

Data are presented as n (% per column). Comparisons between ER-positive and ER-negative patients were performed using chi-square tests. ECOG-PS refers to baseline performance status. FIGO staging based on the 2009 classification. Early Stage = IA–IIIB, Advanced Stage= IIIC–IVB.
